# Exploring Jasmonates in the Hormonal Network of Drought and Salinity Responses

**DOI:** 10.3389/fpls.2015.01077

**Published:** 2015-12-01

**Authors:** Michael Riemann, Rohit Dhakarey, Mohamed Hazman, Berta Miro, Ajay Kohli, Peter Nick

**Affiliations:** ^1^Molecular Cell Biology, Institute of Botany, Karlsruhe Institute of Technology, Karlsruhe, Germany; ^2^Plant Breeding Genetics and Biotechnology Division, International Rice Research Institute, Makati, Philippines

**Keywords:** phytohormones, jasmonic acid, abscisic acid, abiotic stress, drought, salinity

## Abstract

Present and future food security is a critical issue compounded by the consequences of climate change on agriculture. Stress perception and signal transduction in plants causes changes in gene or protein expression which lead to metabolic and physiological responses. Phytohormones play a central role in the integration of different upstream signals into different adaptive outputs such as changes in the activity of ion-channels, protein modifications, protein degradation, and gene expression. Phytohormone biosynthesis and signaling, and recently also phytohormone crosstalk have been investigated intensively, but the function of jasmonates under abiotic stress is still only partially understood. Although most aspects of jasmonate biosynthesis, crosstalk and signal transduction appear to be similar for biotic and abiotic stress, novel aspects have emerged that seem to be unique for the abiotic stress response. Here, we review the knowledge on the role of jasmonates under drought and salinity. The crosstalk of jasmonate biosynthesis and signal transduction pathways with those of abscisic acid (ABA) is particularly taken into account due to the well-established, central role of ABA under abiotic stress. Likewise, the accumulating evidence of crosstalk of jasmonate signaling with other phytohormones is considered as important element of an integrated phytohormonal response. Finally, protein post-translational modification, which can also occur without *de novo* transcription, is treated with respect to its implications for phytohormone biosynthesis, signaling and crosstalk. To breed climate-resilient crop varieties, integrated understanding of the molecular processes is required to modulate and tailor particular nodes of the network to positively affect stress tolerance.

## Introduction

During the last century, the Green Revolution led to food security for a rapidly growing global population through an impressive growth of productivity achieved by mineral fertilizers, chemical plant protection, and mechanization. However, the central factor driving the yield increase was genetics. For instance, the reduction of culm length by mutations in DELLA gibberellin-response factors (Peng et al., 1999) substantially reduced losses by lodging that, in rice, can reach up to 40% (Nishiyama, 1986). Although there is still potential for further increases in crop yield, it has also become clear that plant science of the new century must address additional targets: The land amenable to agriculture is limited and crop production is further constrained by land use for biofuels, urbanization, and desertification. The situation is further accentuated by unpredictable patterns of climate change. The case of desperate farmers who, in expectation of high yields, spend their funds for seeds of high-yielding improved cultivars, and then witness crop failure due to altered rain, temperature and light regimes illustrates that crop breeding must integrate additional traits in addition to improved photosynthetic efficiency or optimal partitioning of assimilates to the culm, for example. Altered regimes of abiotic factors lead to alterations in biotic stress factors as a corollary. Crop resilience to biotic and abiotic stress conditions has therefore shifted into the focus of plant research worldwide (for review, see Passioura, 2002). During evolution, the immobile nature of plants has forced them to evolve unique and sophisticated mechanisms to tolerate abiotic stress. The natural variation in those mechanisms can be used to develop more tolerant crop plants. As prerequisite, we have to understand the underlying molecular, biochemical, and physiological aspects of stress tolerance.

Among the abiotic stress factors, the varied osmotic challenges posed by drought, salinity, and alkalinity together account for maximal yield losses in major crops. Water scarcity is probably the most serious constraint for crop quality and productivity among all environmental factors, compromising economic output and human food supply (Roche et al., 2009). Just salinity alone affects approximately 20% of the irrigated lands of the world. In addition, every year a large fraction of agricultural land is oversalted and becomes unusable (Yeo, 1999; Williams, 2001). The costs to agriculture caused by salinity are huge and are expected to increase as further regions are contaminated with salt (Ghassemi et al., 1995). For instance, deposition of toxic salt sediments and sea intrusion in tsunami-affected areas of the Maldives damaged 70% of agriculture land, destroyed some 370,000 fruit trees, and affected around 15,000 farmers, with estimated costs at around AU$ 6.5 million (FAO, 2005). Unfortunately, salinity is a man-made problem to some extent, caused by agricultural practices such as land clearing and the replacement of perennial vegetation with annual crops and irrigation schemes using salt-rich irrigation water or having insufficient drainage (Munns, 2002). The impact of drought in terms of yield, economy and negative effect on society is even more substantial (Kantar et al., 2011). An FAO report estimated that drought wrought irrevocable negative implications on two billion and killed 11 million people during the last century, more than any other hydro-meteorological hazard (FAO, 2013).

Although in general perceived as mere water scarcity, osmotic stress in reality represents a complex syndrome comprising at least three components that can occur either individually or in different combinations: water scarcity stress (drought), ionic stress (salinity), and nutrient-depletion stress (alkalinity). Adaptation to the three osmotic stresses requires cellular and physiological responses that must be at least partially different depending on the dominating stress component. For instance, drought can primarily affect cell turgidity causing growth arrest and stomatal closure, resulting in photosynthetic imbalance, and impaired redox homeostasis. Salinity can, in addition, impair ionic homeostasis. Thus, adaptation to salinity not only has to reinstall turgidity, but also has to reinstall the equilibrium between important ions such as sodium and potassium. Specific adaptive responses must be triggered by specific signaling cascades as well, involving specific molecular components. However, the number of molecular players that convey stress signals in plants is rather limited and many of these molecular players are shared between different stresses (Ismail et al., 2014b). One model to explain this specificity achieved by a limited set of mostly common factors conceptualizes particular spatiotemporal patterns (so called signatures) of these overlapping signals and the signaling pathways (Ismail et al., 2014b).

A proof of concept for this signatures model, is provided by stress induced calcium patterns. By means of aequorin-reporter plants, different stress factors were shown to produce different temporal signatures of calcium (Knight et al., 1991, reviewed in McAinsh and Hetherington, 1998). The fact that signatures differ between different stresses, does not prove *per se* that these signatures are causative for signal specificity. A functional proof requires manipulating the signatures, which is far from trivial. In case of calcium, this was successfully achieved in guard cells by rhythmic incubations with calcium-containing and calcium-free buffers, and this artificial calcium signature allowed rescuing the deficient stomatal closure in the *det3* mutant of *Arabidopsis* (Allen et al., 2000). Similar signatures seem to act also for other stress signals. For instance, the specificity of reactive oxygen species (ROS) as signals in the processing of drought and salinity stress seems to depend on their subcellular distribution (reviewed in Miller et al., 2010). Likewise, the interaction of jasmonate signaling with other signal chains converging at the proteasome can generate specific outputs (reviewed in Kazan and Manners, 2008). As common theme in these examples, the specificity of signaling seems to stem from specific combinations of fairly general primary signals. For instance, while drought, salt, and cold stress will all activate calcium influx, the “physiological meaning” of this influx is modulated by different, stress-quality specific second messengers to yield different responses (Xiong et al., 2002). This combinatorial model predicts that there exists something like a “grammar of stress signaling,” and when we want to understand, how plants can discriminate the three components of osmotic stress and even specific combinations of them, we have to decipher this “grammar” in the first place.

As a proof of concept, temporal signatures have been dissected for salinity stress in grapevine cells (reviewed Ismail et al., 2014b). Input is either through a (mechanosensitive) calcium influx channel at the membrane, which often acts in concert through the membrane-located NADPH-oxidase Respiratory burst oxidase Homolog (RboH), generating apoplastic ROS. Transduction is conveyed by calcium-binding proteins (CDPKs, calcineurins), a MAP-Kinase cascade, and jasmonates [oxophytodienoic acid (OPDA), jasmonic acid (JA), JA-Isoleucine conjugate (JA-Ile), methyl jasmonate (MeJA)]. Depending on the relative temporal patterns of these upstream signals, the cellular responses were qualitatively different. In one case adaptive responses such as activation of enzymatic antioxidants, osmoprotectants or ion channels resulted in cellular adaptation, whereas in the other case, temporal shifts of early signaling events culminated in necrotic death, which for a cell is a fatal outcome, but for a plant may be adaptive, because it provides a strategy to exclude noxious salt by abscission of a leaf that is thus sacrificed for the sake of the entire plant.

The synthesis, modification, and signaling of jasmonates has been reviewed comprehensively by Wasternack and Hause (2013), and these authors have also extensively treated the role of this pathway for the plant responses to different stress factors. The current review will therefore focus on the role of temporal jasmonate signatures for the adaptive response to osmotic challenges (drought and salinity stress). We address possible mechanisms ensuring specificity for these partially similar stress conditions that on the other hand require partially different responses. In particular, we show that dynamic feedback of jasmonate signaling upon jasmonate synthesis along with different ramifications in synthesis and modification of JA are relevant to constrain this potentially dangerous stress signal in a manner that is tuned with activation of other pathways, prominently abscisic acid (ABA) signaling.

## The Primary cause for Specificity must be Searched in Different Channels of Perception

Modulations in the phytohormonal levels and signaling status in response to abiotic stress have been intensively studied for decades, with often contradictive results, where upregulation of a given hormone was found to confer stress adaptation in one case, but was found to impair survival in a different case. These discrepancies show already that phytohormones apparently do not act as early transducers of stress signals, but rather seem to act as integrators of different upstream signals. Therefore, before we will deal jasmonates themselves, it is important to have a look on these upstream signals.

The actual input for *drought stress* signaling is certainly mechanical load of the membrane. The osmotically caused loss of turgidity will affect membrane tension, and this can be perceived through changes in the activity of mechanosensitive ion channels, a mechanism that was developed early in evolution and functions already in prokaryotic cells (reviewed in Kung, 2005). In plant cells, such mechanosensitive channels drive an influx of calcium. Contrarily, the calcium output caused by mechanical challenge of the membrane is also used to perceive a range of stress factors different from osmotic stress such as touch, gravity, wounding, or cold (reviewed in Nick, 2011). The molecular nature of these channels has remained elusive for decades. The recent discovery of the calcium channel OSCA1 from *Arabidopsis thaliana* that is gated by hyperosmotic stress (Yuan et al., 2014) might mean that a central player for the perception of osmotic challenge of the membrane has been identified. The influx of calcium can be transduced through calcium dependent kinases into activation of the NADPH oxidase RboH generating apoplastic singlet oxygen, such that calcium influx is followed (with some delay) by a transient oxidative burst (Dubiella et al., 2013). At the cellular level, adaptation is brought about by production of compatible osmolytes that will help to reinstall turgidity. Also, a well-known response is the synthesis of late-embryogenesis abundant (LEA) proteins that will prevent protein precipitation (Tunnacliffe and Wise, 2007). At the organismal level, rapid closure of stomata will reduce additional loss of water (for review Xoconostle-Cázares et al., 2011).

For *salinity stress*, the osmotically induced Ca influx is accompanied by a second factor, ionic stress. In fact, sodium ions can pass the plasma membrane by non-selective cation channels (NSCCs). A comparison of two grapevine cell lines that differ in salt tolerance revealed that efficient adaptation correlated with a more rapid uptake of sodium into the cytoplasm indicating that the concomitant increase of cytosolic calcium and sodium might act as a signal triggering salinity adaptation (Ismail et al., 2014a). The adaptive salt overly sensitive (SOS) module cannot only extrude sodium from the cytoplasm, but also links cytosolic sodium with calcium signaling (reviewed in Ismail et al., 2014b). Although some of the adaptive responses to salinity are shared with drought stress (reviewed in Hasegawa et al., 2000), such as induction of osmolytes, accumulation of LEA proteins, or stomatal closure, others are specific for salinity. For instance, sodium can be extruded by the SOS1 exporter (Munns and Tester, 2008), or it can be sequestered into the vacuole through the NHX1 transporter system (Munns and Tester, 2008), which allows to restore turgidity and thus to reinstall growth.

*Alkalinity stress* represents an accentuated version of salinity stress and is of vast agronomic impact with worldwide almost 1000 million hectares being affected (Rao et al., 2008). Although it is known that alkaline sodium stress has much harsher effects as compared to equimolar salinity at neutral pH (Wang et al., 2011), the molecular signals as well as the adaptive mechanisms are far from understood. In addition to osmotic challenge and ionic stress, alkalinity causes the precipitation of important nutrients including phosphates and metallic micronutrients, and also destroys the cellular structure of the roots (Li et al., 2009). Under physiological conditions, the apoplast is actively maintained at a slightly acidic pH of around 5.5 by proton ATPases localised in the plasma membrane and this activity is essential to sustain cell expansion growth (Haruta et al., 2010). Under alkaline conditions, this mechanism is interrupted. Moreover, the activity of osmotically induced calcium influx is expected to be impaired, because calcium enters the cell by cotransport with protons (which allows to conveniently monitoring this influx as transient alkalinization of the apoplast). As a second effect, the superoxide anions that are generated by the NADPH oxidase RboH to a certain extent even under normal conditions will not be dissipated due to the absence of protons as electron acceptors, leading to an accentuated stress-induced oxidative burst. Adaptation to alkalinity must involve mechanisms that transcend conventional salinity responses, a point that so far has not been appropriately considered in breeding programs (Bui, 2013). For instance, in addition to sequestering sodium in the vacuole, and quelling the accentuated oxidative burst, adaptation to alkalinity would also require powerful buffering of the apoplast, which might either be achieved through upregulation of proton ATPases or through secretion of organic acids.

The comparison of the three aspects of osmotic stress illustrates that each specific condition requires a specific adaptive response, which seems to be determined by specific equilibria between different stress inputs. This adaptive response is costly, however. For instance, stomatal closure will reduce water loss by transpiration, but it will also reduce photosynthetic efficiency and lead to secondary photooxidative stress caused by unbuffered electron transport in the thylakoid (Pinheiro and Chaves, 2011). Similarly, the synthesis of polyamines binds precious bioavailable nitrogen (Alcázar et al., 2006). Therefore, these adaptive responses have to be carefully adjusted to growth and development. It is this adjustment, where phytohormonal signaling links with stress adaptation. The jasmonate signaling system seems to act as a hub, where different inputs are processed to yield an appropriate adaptive response.

**FIGURE 1:**
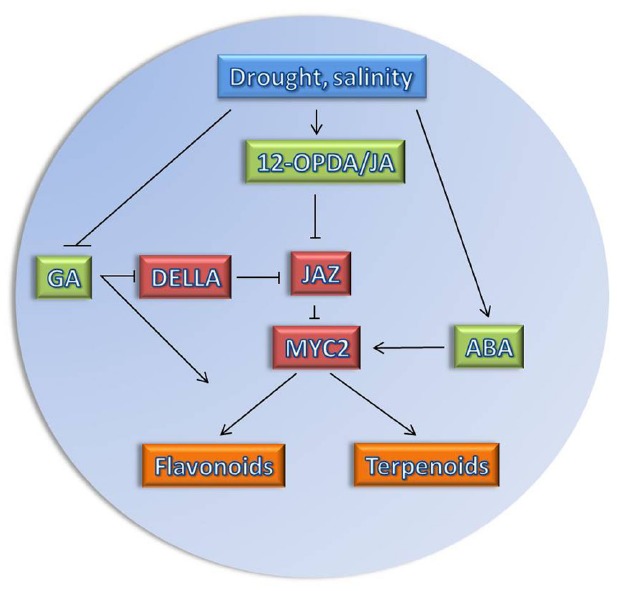
**Model of JA network in response to drought/salinity stress.** Osmotic stress affects the biosynthesis of the plant hormones (highlighted in green), jasmonic acid (JA), 12-oxo-phytodienoic acid (12-OPDA), and abscisic acid (ABA) positively (Takeuchi et al., 2011; Hazman et al., 2015), while the biosynthesis of GA is repressed (Colebrook et al., 2014). Changes in hormonal levels affect several important key regulatory proteins (highlighted in red). The JAZ repressor is degraded when JA is produced, such that the transcription factor MYC2 will be activated. MYC2 could work as signaling hub (Kazan and Manners, 2013) as it is also positively regulated by ABA. Due to repression of GA biosynthesis, DELLA proteins, repressors of GA signaling, will accumulate. DELLA has been shown to interact physically with JAZ proteins and hence can relieve MYC2 from JAZ repression (Hou et al., 2010). MYC2 activates secondary plant metabolic pathways such as flavonoids and terpenoids (highlighted in orange) which are required for plant adaptation to stress conditions.

The following sections investigate the role of jasmonates for drought and salinity signaling and attempt to dissect the interaction of jasmonate signaling with the signaling triggered by ABA. Jasmonate biosynthesis utilizes different metabolites with potentially different biological activity. A complex feedback regulation of this pathway allows for each node to function in either direction to process several inputs with ample ramifications into different outputs as required for a signaling hub (Figure 1). Such a system acts in a highly non-linear fashion, which means that even subtle modulations in the relative activities of individual components of this hub can result in a qualitatively different output. This also means that breeding of stress-tolerant crops might not require drastic genetic changes. Slight, but targeted shifts in the relative activities of jasmonate signaling components might be worth exploring. This is particularly interesting in view of the independent evolution of salinity tolerance in some of the 3000 grasses (Bennett et al., 2013), which may be suggestive of perturbances in a limited number of pathways, but at different nodes. Thus, there may be more than one road leading to Rome.

## Placing Jasmonates into the Drought and Salinity Signaling Cascade

Both drought and salt stress are multidimensional in nature and affect plants at various levels of their organization (Yordanov et al., 2000). Therefore, the effects of stress are often observed at morpho-physiological, biochemical and molecular levels, such as growth inhibition (Bahrani et al., 2010), enhanced production of compatible organic solutes (Sánchez-Díaz et al., 2008; DaCosta and Huang, 2009), changes in the content of phytohormones (Perales et al., 2005; Seki et al., 2007; Huang et al., 2008; Dobra et al., 2010; Kohli et al., 2013), or altered expression of stress responsive-genes (Xiong and Yang, 2003; Yamaguchi-Shinozaki and Shinozaki, 2005; Huang et al., 2008). Changes tissue water status trigger some of these responses directly, while many others are brought about by plant hormone-dependent signaling (Chaves et al., 2003). The tolerance/adaptation response of plants to unfavorable environmental conditions strongly depends on chemical signals/secondary metabolites that are orchestrated by plant hormones in a complex balance between tolerance and growth (Sreenivasulu et al., 2012, Figure 1). It has been known previously that hormones do not function in discrete pathways, but rather influence each other at different levels (i.e., biosynthesis or signaling) to control environmental and developmental signaling pathways (Gray, 2004). This will create a signal transduction network that can integrate different inputs into a comprehensive output culminating in physiological adaptation of the plant to stress.

**FIGURE 2:**
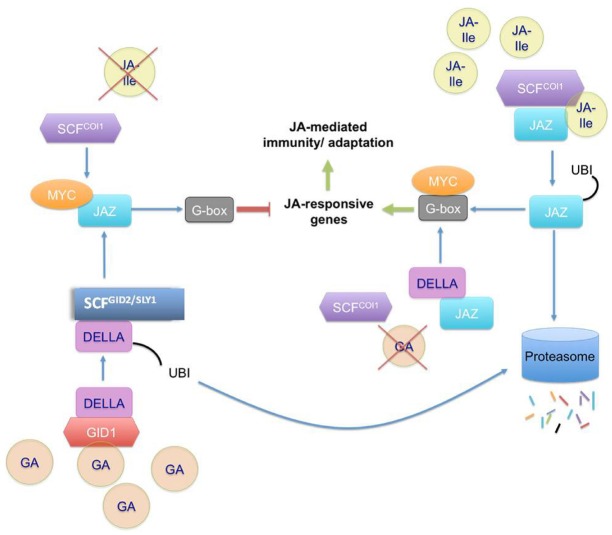
**Schematic representation of JA signaling activation and repression in the presence of JA and GA.** JA-Ile mediates the activation of JA responsive genes by attaching to JAZ proteins and F-box COI1 complexes; which in turn become ubiquitinylated and are committed to the degradation pathway. MYC transcription factors 2, 3, and 4 are then free to attach to the G-box domain and activate the transcription of the JA-responsive genes. In the absence of JA-Ile, JAZ proteins bind to the transcription factors and restrict gene expression. A similar mechanism is observed in the presence/absence of GA. This pathway is mediated through DELLA proteins. In the presence of GA, the repression is activated. GA binds to the receptor GID1, then DELLA can bind to the hormone-receptor complex. Subsequently, DELLA binds to GID2/SLY1, the F-box protein in SCF-GID2/SLY1 which promotes DELLA ubiquitination. That leaves JAZ proteins free to bind to MYC transcription factors and the JA-responsive genes cannot be transcribed. On the other hand, the absence of GA means that DELLA are not sent to the degradation pathway and bind to JAZ. JAZ cannot bind to MYC transcription factors, which are available to bind the G-box and activate the transcription of JA-responsive genes.

A key role in this hormonal network is played by the plant hormone ABA. Its function in the control of stomata closure and the responses to abiotic stress is well-established and has been intensively studied since decades (for review, see Mittler and Blumwald, 2015). Drought stress or high salinity cause accumulation of ABA in plants and extensive changes in gene expression (Shinozaki and Yamaguchi-Shinozaki, 2007). ABA signaling triggered by receptors in the plasma-membrane as well as the cytoplasm, has been intensively studied in guard cells (for review, see Mittler and Blumwald, 2015). Subsequent signaling increases the concentration of cytosolic Ca^2+^ due to the activation of calcium channels in the endoplasmic reticulum, which further activates or inhibits ion channels in the plasma membrane. As a result of ion fluxes, water potential in the apoplast decreases and water flows out of the cell leading to a lower turgor of guard cells and closure of stomata. Due to this central function of ABA for the regulation of stomatal opening and closure and the control over other stress adaptive mechanisms, this hormone is very important for the response to abiotic stress. However, usually changes in one hormonal pathway affects the pathways of other hormones and expectedly other hormones, especially those related to stress and growth responses, contribute to the overall response of the plant. One of these hormonal pathways currently attracting a lot of attention, is jasmonate signaling (JAs), conveyed by JA and its derivatives. JAs constitute a group of fatty acid-derived compounds that play prominent roles in coordinating inducible defense responses leading to increased tolerance to insect pests and necrotrophic pathogens (for review, see Wasternack and Hause, 2013). JAs are also required for specific steps of plant development like reproduction or photomorphogenesis (for review, see Svyatyna and Riemann, 2012). Biosynthesis, perception and action of JAs have been extensively studied. On the contrary, inactivation/removal mechanisms have remained elusive for a long time, but have been elucidated recently (Heitz et al., 2012; Aubert et al., 2015). In sharp contrast to most other plant hormones, JA must be activated by enzymatic coupling to isoleucine amino acid. The resulting JA-Ile functions as a ligand promoting assembly of a co-receptor complex between the F-box protein COI1 and so-called JA ZIM-domain (JAZ) proteins (Chini et al., 2007; Thines et al., 2007). JAZ proteins are transcriptional repressors that prevent the transcription of target genes under low JA-Ile levels, and are specifically ubiquitinated when JA-Ile accumulates under biotic stress. This is a signal leading to their proteolytic degradation, relieving active transcription of JA-responsive defense genes from repression (Figure 2). JA-Ile is therefore a master switch controlling various aspects of plant immunity/adaptation. Elements under JA control include the synthesis of digestive inhibitors targeting insects, volatile repellents, and many toxic or antimicrobial compounds that lower the performance of pests. Although role for jasmonates for the adaptation to salt stress has been suggested (Fujita et al., 2006), molecular mechanisms of the role of jasmonates for salt or drought stress-signaling are still mostly unclear. The following sections review what is known on how jasmonates contribute to the plant response toward these two intensively studied abiotic stresses, drought and high salinity.

## Jasmonates and Drought Stress

There is a steadily increasing body of evidence for the involvement of jasmonates in drought stress. Barley leaves exposed to simulated drought with sorbitol or mannitol exhibited increased endogenous contents of jasmonates, followed by the transcription of jasmonate-induced proteins (JIPs, Lehmann et al., 1995). A later study also showed that the contents of octadecanoids and JAs were enhanced by sorbitol treatment to a degree, sufficient to initiate JA-responsive gene expression (Kramell et al., 2000). In addition, endogenous JA content increased in maize root cells under drought stress (Xin et al., 1997), and this compound was also able to elicit betaine accumulation in pear leaves (Gao et al., 2004). In some studies, JA has been reported to improve drought tolerance but in others, it has been reported as a negative agent that causes notable reduction in growth and yield, hence the actual role of JA in drought stress remains controversial. Mostly, the observed responses depend on the type of plant and tissue in question, intensity and duration of drought stress and the dosage of JA applied (Lee et al., 1996; Kim et al., 2009). Therefore, a lot of the controversy might actually result from the fact that studies were done under different conditions, e.g., in various developmental stages, tissues, and with different stress regimes. This might be linked with the fact that also the degree of drought tolerance strongly depends on developmental stage in most plant species (Reddy et al., 2004; Rassaa et al., 2008).

### Exogenous Jasmonates can Increase Drought Tolerance

Several reports suggest that exogenous application of jasmonates ameliorates the response of plants to drought stress. It has been reported that exogenous application of JA or MeJA increased the antioxidative capacity of plants under water stress (Bandurska et al., 2003). In the same context, other studies also showed that JAs play an important role in signaling drought-induced antioxidant responses, including ascorbate metabolism (Li et al., 1998; Ai et al., 2008). It has been observed that exogenous JA is effective in protecting plants from drought-induced oxidative damage as it enhances the activity of antioxidant enzymes (Nafie et al., 2011). It is also hypothesized that MeJA could ameliorate water stress tolerance in banana by regulating the growth, proliferation rate, proline accumulation, chlorophyll levels, tissue water status, oxidative stress, and membrane lipid peroxidation (Mahmood et al., 2012).

Another study was conducted by Anjum et al. (2011) in soybean (*Glycine max* L. Merrill) to explore the role of exogenous MeJA application in alleviating the adversities of drought stress. Soybean plants were grown under normal conditions until blooming and then were subjected to drought by withholding water followed by foliar application of MeJA. From the observed data, it was noticed that drought stress substantially lowered the yield and yield-related traits, whereas it accelerated the peroxidation of membrane lipids. Nonetheless, considerable increase in the activity of antioxidant enzymes such as superoxide dismutase (SOD), peroxidase (POD), and catalase (CAT), and in proline, relative water contents (RWC) along with simultaneous decrease in membrane lipid peroxidation was observed when the drought stressed plants were treated by MeJA. These beneficial effects led to significant improvement in yield and harvest index under drought. Interestingly, MeJA application was also promotive under well-watered conditions. These results suggested that by modulating the peroxidation of membrane lipids and antioxidant activities, MeJA improved the drought tolerance of soybean.

### Which Jasmonates Contribute to Drought Stress Signaling?

Jasmonates comprise a diverse group of JA derivatives (for review, see Wasternack and Hause, 2013), including its biosynthetic intermediate, 12-OPDA, which is capable of activating specific signaling events (Taki et al., 2005). Recent data indicate that 12-OPDA might be the jasmonate derivative which is mainly functional in the drought response.

De Domenico et al. (2012) measured the expression of key genes involved in oxylipin metabolism by quantitative PCR on samples from stressed and non-stressed roots of a drought-tolerant and a drought-sensitive chickpea variety. In their study, they demonstrated that the drought tolerant variety reacts to drought with sustained and earlier activation of a specific lipoxygenase (*MtLOX1*), two hydroperoxide lyases (*MtHPL1* and *MtHPL2*), an allene oxide synthase (*MtAOS*), and an oxo-phytodienoate reductase (*MtOPR*). Over-expression of these genes correlated positively with the levels of major oxylipin metabolites from the allene oxide synthase (AOS) branch of the pathway, which finally leads to the synthesis of jasmonates. The roots of the tolerant variety accumulated higher levels of JA, its precursor OPDA and the active JA-Ile, suggesting a role of jasmonates for drought tolerance in chickpea.

Savchenko et al. (2014) identified that drought led to a block in the conversion of 12-OPDA to JA and further revealed that 12-OPDA was the functional convergence point of oxylipin and ABA biosynthesis pathways, to control stomatal aperture in plant-adaptive responses to drought stress. They used three *A. thaliana* ecotypes to demonstrate that wounding induced both 12-OPDA and JA levels, whereas drought induced only the precursor 12-OPDA. This implicated the AOS branch of the oxylipin pathway as a critical node. Levels of ABA were also mainly enhanced by drought and little by wounding. To explore more about the role of 12-OPDA in plant drought responses, they also generated a range of transgenic lines and exploited existing mutant plants that differ in their levels of stress-inducible 12-OPDA, but displayed similar ABA levels. The plants which were producing higher 12-OPDA levels exhibited enhanced drought tolerance and reduced stomatal aperture. Furthermore, on exogenous application of ABA and 12-OPDA, whether individually or combined, stomatal closure was promoted in the ABA and AOS biosynthetic mutants, albeit most effectively when combined. Using tomato (*Solanum lycopersicum*) and *Brassica napus*, they verified the potency of this combination in inducing stomatal closure in plants other than *Arabidopsis*. They concluded that drought was a stress signal that uncoupled the conversion of 12-OPDA to JA and also revealed 12-OPDA as a drought-responsive regulator of stomatal closure functioning most effectively together with ABA.

### Jasmonates Contribute to Regulation of Stomatal Closure

Based on its accumulation during drought stress and its positive regulatory role in stomatal closure, JA has been proposed as important player for stomatal closure during drought stress (Gehring et al., 1997; Suhita et al., 2003, 2004). Soybean leaves under water stress showed a 15% loss of fresh weight and accumulated fivefold more JA within 2 h, but the level of JA declined to that of control plants by 4 h (Creelman and Mullet, 1995). MeJA-mediated stomatal closure has been related to cytoplasmic alkalinization in guard cells, production of ROS (via AtRboHD/F) and NO, and activation of K-efflux (Evans, 2003), as well as slow anion channels (Gehring et al., 1997; Suhita et al., 2003, 2004; Munemasa et al., 2007). All these effects are similar to those of ABA, thereby suggesting an overlapping use of signaling components for stomatal closure. This idea is also supported by observations made in the ABA hyposensitive *ost1* mutant, which turned out to be less sensitive to MeJA with respect to stomatal closure. Moreover, the MeJA insensitive mutant *jar1* displays reduced stomatal closure in response to ABA (Suhita et al., 2004).

### Do ABA and JA Act Synergistically in Drought Stress Signaling?

Abscisic acid plays a key role in plant adaptation to adverse environmental conditions including drought stress. However, molecular, genetic and genomic analyses suggested that in addition to ABA-dependent pathways, ABA-independent regulatory systems are involved in stress-responsive gene expression (Bray, 1997; Shinozaki and Yamaguchi-Shinozaki, 1997, 2000; Riera et al., 2005). Induction of ABA synthesis is one of the fastest phytohormonal responses of plants to abiotic stress, thereby triggering ABA-inducible gene expression (Yamaguchi-Shinozaki and Shinozaki, 2006), causing stomatal closure, and hence reducing water loss via transpiration (Wilkinson and Davies, 2010), which will eventually restrict cellular growth. During the adaptive responses of plants to environmental stresses, the overlap between hormone-regulated gene expression profiles suggests the existence of a complex network with extensive interactions between the different hormone signaling pathways. In order to examine a crosstalk between ABA and JA signal transduction *Arabidopsis* ABA-insensitive (*ost1-2*) and MeJA-insensitive (*jar1-1*) mutants were studied for the participation of ABA and JAs in stomatal closing (Suhita et al., 2004). The authors investigated changes of cytoplasmic pH and ROS production in response to ABA or JA, and the mutants were used to assess the respective roles of the mutated genes in ABA or JA signaling pathways leading to stomatal closure. The modulation of Ca^2+^ions was induced by both, ABA and JA. However, the primary actions of ABA and JA in the plasma membrane appear to be different: JA targets the Ca^2+^ channels whereas ABA activates effectors in the plasma membrane (e.g., phospholipase C and D). However, at the level of intracellular Ca^2+^, both signal transduction pathways converge. Intracellular Ca^2+^ level is modulated to a much greater extent by JA than by ABA.

It is well established that JA biosynthesis is induced by stress conditions such as wounding and herbivory (Wasternack, 2007), but many JA-associated signaling genes are also regulated by drought stress (Huang et al., 2008). It has been shown that JA interacts with ABA-regulated stomatal closure by increasing Ca^2+^ influx, which activates a CDPK-dependent signal cascade. Treatment of turgescent, but excised *Arabidopsis* leaves with either ABA or MejA resulted in a reduction of stomatal aperture reduction within 10 min (Munemasa et al., 2007). Through the inhibition of ABA biosynthesis by chemical inhibitors or in ABA-deficient mutants, the MeJA-induced Ca^2+^ oscillations in guard cells are suppressed, and also stomatal closure is impaired (Hossain et al., 2011). Therefore, it has been postulated that MeJA-mediated regulation of stomatal closure interacts with ABA-mediated regulation of Ca^2+^ signal transduction pathways. Studies related to the interactions of ABA with MeJA in guard cells show that both hormones induce the formation of ROS and NO, and also that both are present at reduced concentrations in MeJA-insensitive plants (Munemasa et al., 2007).

The combined effect of ABA and JA for acclimation to stress in *Arabidopsis* may be mediated by an extensive genetic reprogramming to finally reach a new homeostasis (Harb et al., 2010). These authors suggested that endogenous JA together with high ABA level are sufficient to stimulate the preparatory response needed for drought acclimation (e.g., stomatal closure and cell wall modification) during the early stages of moderate drought (30% field capacity). Probably, JA is not required at high concentration under drought stress, and plant growth would be even negatively impacted by high concentrations. For example, the JA-insensitive *coi1* and *jin1*, mutants of *Arabidopsis* were found to be significantly resistant (or insensitive) to moderate drought stress. Biomass accumulation as compared to wild type under drought did not differ from the well-watered control. These results were in agreement with studies showing that the JA-mediated inhibition of seedling and root growth is suppressed in the *coi1* mutant (Xie et al., 1998). Harb et al. (2010) suggested that in the absence of JA signal perception, the developmental program for acclimation to stress, i.e., reduced growth is not switched on. Thus, the signaling pathways for plant growth under prolonged drought might converge on the down-regulation of JA biosynthesis to minimize its inhibitory effect on plant growth, thus establishing a new state of homeostasis by the acclimation process.

The proposed overlap between the JA and ABA stress signaling cascades (Fujita et al., 2006; Harb et al., 2010) has stimulated the search for transcription factors and kinases as promising candidates for common players in this interaction. For example, the transcription factor AtMYC2 plays a role in multiple hormone signaling pathways. From the genetic analysis of the jasmonate-insensitive *jin1* mutant, it was revealed that *JIN1* is allelic to *AtMYC2*, which was first identified as a transcriptional activator that is involved in the ABA mediated drought-stress signaling pathway (Abe et al., 2003). Downstream targets, such as *RD22*, a gene responsive to dessication and salt stress, is activated by both, AtMYC2 and R2R3MYB-type, transcription factors. Similarly, expression of *RD26* is induced by hydrogen peroxide, pathogen infections, and JA, as well as by drought, high salinity and ABA treatment (Fujita et al., 2004, 2006; Harb et al., 2010). In addition, protein phosphorylation and dephosphorylation by kinases and phosphatases, respectively, can significantly affect the regulation of morpho-physiology and gene expression associated with JA-dependent root growth. However, enzymes phosphorylating or dephosphorylating *AtMYC2* have not been identified yet (Kazan and Manners, 2008).

To specifically address the crosstalk of ABA and JA at the whole plant level, the tomato ABA-biosynthetic mutant *sitiens* was used. When the petioles of *sitiens* were incubated in JA, they did not show any indications of stomatal closure as assessed by gas-exchange measurements; however, when pre-incubated with ABA, petioles showed stomatal closure in response to JA (Herde et al., 1997). This suggested that in tomato, ABA was required for the JA-mediated stomatal regulation. In soybean, it was observed that exogenous application of MeJA did not affect endogenous ABA levels. However, water stressed barley seedlings that had been pre-treated with JA showed more than fourfold accumulation of ABA in comparison to the control. This clearly suggested a role for JA in ABA biosynthesis under water stress conditions (Bandurska et al., 2003). MeJA regulates numerous drought-responsive genes (Huang et al., 2008), many of which are also regulated by ABA with similar expression kinetics (Nemhauser et al., 2006; Huang et al., 2008). Overall, all these data support the concept of common signaling components for ABA and MeJA, including nitric oxide (NO; for review, see Daszkowska-Golec and Szarejko, 2013).

### Involvement of Jasmonates in the Drought Response in Rice

Substantial information exists about the roles of phytohormones under drought in model plants such as *Arabidopsis*. A current challenge is to transfer this knowledge to other, economically more relevant, plant species. Two examples of investigations performed in rice are presented as case studies.

Seo et al. (2011) used a functional genomics approach that identified a basic helix-loop-helix domain gene (*OsbHLH148*) that conferred drought tolerance as a component of the jasmonate signaling module in rice. They found that *OsbHLH148* transcript levels were rapidly increased by treatment with MeJA or ABA, as well as by abiotic stresses including dehydration, high salinity, low temperature and wounding. Over-expression of *OsbHLH148* in rice conferred tolerance to drought stress. Expression profiling followed by DNA microarray and RNA gel-blot analyses of transgenic versus wild type rice identified genes that were up-regulated by over-expression of *OsbHLH148*. These genes included *OsDREB* and *OsJAZ*, genes involved in osmotic stress responses and jasmonate signaling, respectively. *OsJAZ1*, a rice ZIM domain protein, interacted with *OsbHLH148* in yeast two-hybrid and pull-down assays and it interacted with the putative OsCOI1 only in the presence of coronatine. Furthermore, the OsJAZ1 protein was degraded by rice and *Arabidopsis* extracts in the presence of coronatine, and its degradation was inhibited by MG132 which is a 26S proteasome inhibitor, suggesting 26S proteasome-mediated degradation of OsJAZ1 via the SCF^OsCOI1^ complex. These results suggested that OsJAZ1 was a transcriptional regulator of the OsbHLH148-related jasmonate signaling pathway leading to drought tolerance, suggesting that OsbHLH148, OsJAZ, and OsCOI1 constitute a signaling module in rice.

Another study by Kim et al. (2009) demonstrated that constitutive overexpression of the *Arabidopsis JASMONIC ACID CARBOXYL METHYLTRANSFERASE* gene (*AtJMT*) in rice increased the levels of MeJA by sixfold in young panicles in rice. Grain yield was greatly reduced due to lower number of spikelets and lower grain-filling rate as compared to non-transgenic (NT) controls. The number of spikelet organs, including the lemma/palea, lodicule, anther, and pistil were altered in these transgenic plants. The loss of grain yield and alteration in spikelet organ numbers were reproduced by treating NT plants with exogenous application of MeJA, thereby indicating that it was the increased levels of MeJA in the AtJMT transgenic rice panicles, that was responsible for the inhibition of spikelet development. Interestingly, in young NT panicles upon exposure to drought conditions, MeJA levels were increased by 19-fold resulting in a similar loss of grain yield. ABA levels were increased by 1.9- and 1.4-fold in the transgenic and drought-treated NT panicles respectively. Increased levels of ABA in the AtJMT transgenic panicles grown in non-drought conditions suggests that it is MeJA, and not drought stress, which induces ABA biosynthesis under drought. A microarray strategy identified seven genes commonly regulated in the AtJMT transgenic and drought-treated NT panicles. Two of these genes, namely *OsJMT1* and *OsSDR* (for short-chain alcohol dehydrogenase), participate in rice in the biosynthesis of MeJA and ABA, respectively. Overall, these results suggested that plants produce MeJA during drought stress, which in turn stimulates the production of ABA, leading to a loss of grain yield.

The two examples above establish the importance of further studies exploring the role of JAs in combating drought stress in rice. Similar studies in other cereals may also be helpful in delineating the role of JA individually or in combination with other phytohormones toward understanding the plant response to drought and toward generating more resilient cereal plant varieties.

## Jasmonates and Salt Stress: A Relation not Easy to be Figured Out

Salinity stress is at least as complex as drought stress. Initially, it mainly triggers three harmful effects, (i) osmotic stress (reduced water uptake),(ii) specific ion toxicity stress (mainly Na^+^ ad Cl^–^), and (iii) oxidative stress by uncontrolled production of ROS, including superoxide radicals (O_2_), hydrogen peroxide (H_2_O_2_), and hydroxyl radicals (OH^–^). These ROS can cause oxidative damage to proteins, enzymes, DNA and RNA (Pessarakli, 2001; Sharma et al., 2012), but can also act as important stress signals. After their role in osmotic stress has been discussed above, now the role of jasmonates in the context of ion toxicity and oxidative stress will be addressed.

Several reports investigated the involvement of JA in salt stress. On the one hand, application of exogenous JAs diminished the damage by salinity in soybean (Yoon et al., 2009) and rice (Kang et al., 2005). On the other hand, the level of endogenous JAs increased under strong salt stress in rice roots (Moons et al., 1997) and in tomato (Pedranzani et al., 2007) suggesting that accumulation of JAs could also protect against salt stress. Nevertheless, it is not possible to draw a general connection between high levels of JA and adaptation. For example, a comparison of two grapevine cell lines differing in their salinity tolerance revealed that the accumulation of JA and JA-Ile was more pronounced in the sensitive *Vitis riparia* than in the salt-tolerant *Vitis rupestris* (Ismail et al., 2012, 2014a). Also, JA was induced after osmotic stress but not after salt stress in barley segments (Kramell et al., 2000) and rice seedlings (Takeuchi et al., 2011). Recent evidence suggests that alterations in the level of JAs affect salinity tolerance in rice. Kurotani et al. (2015) demonstrated that overexpression of *CYP94*, a gene encoding a catabolic enzyme inactivating JA-Ile, results in improved salt tolerance. Suppression of OsJAZ9, a repressor of JA signaling, produced higher sensitivity to JA and an increased sensitivity to salt (Wu et al., 2015). Conversely, rice mutants of the JA biosynthesis enzyme AOC, *hebiba*, and *cpm2*, showed an improved salt tolerance (Hazman et al., 2015). Whether it is the lack of JA or JA-Ile, or whether it is the absence of their precursor 12-OPDA which causes this tolerance, remains to be elucidated. The discrepancies with respect to the role of JAs for salt tolerance indicate that it may not be the presence or absence of JAs that decides the kind of response to salinity, but their timing and control, which may be more important (Ismail et al., 2014b).

### Evidence for the Involvement of Jasmonates in the Uptake of Sodium Ions

In order to cope successfully with high salinity stress, it is necessary for plants to reduce the accumulation of sodium ions into the photosynthetic tissue. This holds especially true for glycophytes to which many important economic crops belong. Currently, it is not clear, whether and how extra- or intracellular sodium ions are sensed, there is no evidence for a receptor of sodium ions in *sensu stricto* (Zhu, 2007). Even though the molecular identity of Na^+^ sensors has remained elusive, the plasma membrane Na^+^/H^+^ antiporter SOS1 might be a probable candidate (Shi et al., 2000). The transport activity of SOS1 is essential for sodium efflux from *Arabidopsis* cells but additionally, its long cytoplasmic tail can bind Na^+^ and might therefore confer sodium sensing (Conde et al., 2011). The JA biosynthesis genes *ALLENE OXIDE SYNTHASE* (*AOS*) and *ALLENE OXIDE CYCLASE* (*AOC*) have been reported to be highly expressed in response to salinity in a SOS-dependent manner (Gong et al., 2001), suggesting a shared pathway. However, the exact link between jasmonates and SOS1 on the level of signaling and functional interactions is far from clear.

It is well known that the plasma membrane around the root hair epidermal cells is responsible for the influx of the largest portion of sodium ions into plant cells at the soil–plant interface. Several candidate genes have been reported as responsible for Na^+^ uptake in plants including NSCCs, high-affinity K^+^ transporters (HKTs), and low-affinity cation transporter (LCT1; Schachtman et al., 1997; Apse and Blumwald, 2007; Craig Plett and Møller, 2010). Additionally and equally important, controlling potassium supply during salinity stress is the main key for survival as the K^+^/Na^+^ ratio should be kept as high as possible for avoiding metabolic failure due to sodium toxicity (Kronzucker et al., 2013). Uptake of sodium ions into rice depends on jasmonates (Hazman et al., 2015), since the *aoc* mutants *hebiba* and *cpm2* accumulated significantly less sodium ions in their shoots, indicating that selective transporters, presumably located in the Casparian strip in the root, might be regulated by jasmonates. It is likely that this trait vary between species and even between cultivars within the same species, which might be one of the reasons, why salt tolerant and salt sensitive cultivars can accumulate different levels of JAs in response to salt stress.

## Jasmonate Crosstalk to other Phytohormones in Abiotic Stress Signaling

In the previous sections, JA crosstalk to ABA and the involvement of protein phosphorylation (e.g., of MYC2) and ubiquitination (e.g., of JAZ repressors) as downstream effects of JA have been mentioned. Although the JA and ABA crosstalk for the regulation of stomatal opening is important, especially under drought, this seems to be not the only crosstalk of JA with other phytohormones or signaling molecules.

Gibberellic acid (GA), generally known as growth hormone, plays also a role in abiotic stress tolerance. The content of GAs may be involved in either growth suppression or promotion under a specific abiotic stress (Colebrook et al., 2014). Crosstalk between GA and JA can be mediated through the DELLA and JAZ proteins which directly interact with each other. This interaction would compete with the JAZ proteins binding to MYC2, a transcription factor activating JA responsive genes, whereby JAZ proteins negatively regulate the JA response. In the presence of JA, the JAZ proteins are committed to proteasome-mediated degradation, while in the presence of GA the DELLA proteins are recruited for proteasome-mediated degradation (Figure 2). Thus, in presence of GA, the JAZ proteins can again bind to MYC2, the alternate partner, and attenuate JA-responsive genes (Boter et al., 2004; Hou et al., 2010). Interestingly, a DELLA gene *RGL3* is transcriptionally upregulated by JA signaling, and the promotor of *RGL3* is a target of MYC2 (Wild et al., 2012). RGL3 physically interacts with JAZ1 and JAZ8, the latter being relatively resistant to JA-mediated degradation (Shyu et al., 2012; Wild et al., 2012). Thus, JA-mediated degradation of JAZ1 releases MYC2 to induce *RGL3*, which in turn binds the non-JA degradable JAZ8 enhancing the MYC2-dependent JA responses (Wild et al., 2012). Further, the GA content determines the extent of degradation of DELLA proteins, and thus the induction amplitude for the JA response, thus linking the two hormone signaling pathways. GA and ABA crosstalk follows a similar pattern under abiotic stress through another DELLA target (Ko et al., 2006; Zentella et al., 2007).

Interaction between auxins including the main natural auxin indole-3-acetic acid (IAA) and JA during plant growth and development have been described including phenomena such as cell elongation, abscission, and tendril coiling, but also wound responses (Saniewski et al., 2002). Recently Du et al. (2013) documented interactions between IAA and JA under drought in rice. Analysis of transcripts related to auxin and JA biosynthesis or signaling showed increased expression of auxin related genes under heat and cold, but a decrease under drought, which was paralleled by corresponding changes of IAA content. However, the content of JA and its associated genes increased under drought and cold but decreased under heat stress. Tiryaki and Staswick (2002) showed that the expression of JA-responsive genes was either repressed or induced by exogenous auxin, suggesting that JA- and auxin-triggered signaling can interact both antagonistically or synergistically. The underlying mechanisms as well as the biological context is far from understood, but one point of convergence might be the GH3 family of acyl acid amido synthetases which contribute to amino acid conjugation of both IAA and JA, which in case of JA generates the active signal, whereas it might be a mechanism of inactivation in case of IAA (Staswick et al., 2005; Khan and Stone, 2007). JA and auxin crosstalk during JA induced lateral root formation involving the ethylene response factor 109 (ERF109) was reported by Cai et al. (2014), and Jiang et al. (2014) recently reported that, in *Arabidopsis*, the WRKY57 acts as a node of convergence in JA and IAA-mediated signaling during JA-induced leaf senescence. *Arabidopsis* JAZ4/8 and IAA29, repressors of JA and IAA signaling, respectively, both competitively bind WRKY57, which is upregulated by IAA, but downregulated by JA. A rice AUX/IAA protein, OsIAA6 has been shown to correlate with drought tolerance in rice (Jung et al., 2015). Thus, JAZ, IAA, ARF, and WRKY genes are known to be act positively on drought tolerance, but the mechanisms of their interaction are yet to be elucidated.

Cytokinins (CK) as further important class of phytohormones driving cell division and meristem formation might also interact with JA signaling. The crosstalk between JA and auxin during meristem formation is well documented (Su et al., 2011), but, so far, there is not much evidence for interplay between JA and CK. However, the two hormonal pathways might be linked antagonistically (Sano et al., 1996; Naik et al., 2002; Stoynova-Bakalova et al., 2008). CK content *in vivo* and application of exogenous CK accelerate the JA-mediated stress response (Sano et al., 1996; Dervinis et al., 2010), while JA application induces the accumulation of CK ribosides (Dermastia et al., 1994). JA biosynthesis is activated in the roots during drought (Poltronieri et al., 2013), and repression of CK biosynthesis and signaling promotes the expansion of the root system, which should act positively on drought tolerance (Werner et al., 2010). Thus, JA and CK signaling/biosynthesis might mainly act in an antagonistic manner.

Similar to JA, also salicylic acid (SA) has been classically associated with biotic stress. However, a combined approach of proteomics and transcriptomics identified common proteins upregulated by JA and SA, associated with oxidative or abiotic stress responses (Proietti et al., 2013). On the other hand, SA can quell the induction of AOS in response to wounding, demonstrating a negative crosstalk from SA upon JA signaling (Harms et al., 1998). The role of SA under abiotic stresses including heat, salt and osmotic stress is well accepted and has been extensively reviewed (Horváth et al., 2007; Pal et al., 2013; Miura and Tada, 2014). The convergence between the JA and SA signaling in *Arabidopsis* was identified as the MAP Kinase 4 (AtMPK4), which negatively regulates the activation of SA- and the repression of JA-mediated defenses under biotic stress (Brodersen et al., 2006). Whether AtMPK4 exerts the same function under abiotic stress remains to be tested, but it is already known that AtMAPK4 is rapidly activated by abiotic stresses (Ichimura et al., 2000). In this regard the role of SA in stomatal closure is noteworthy (Miura and Tada, 2014).

Crosstalk between JA and ethylene is well known for defense against plant pests and pathogens. Once again, however, importance of such crosstalk between JA and ET in abiotic stress has been elaborated only recently (Kazan, 2015). The expression of the *Arabidopsis* ethylene response factor 1 (AtERF1) is activated by both JA and ET (Lorenzo et al., 2003). It has now been reported that synergistic activation of AtERF1 is required for drought and salinity tolerance (Cheng et al., 2013). Constitutive overexpression of AtERF1 additionally produced enhanced tolerance to heat as well. The set of genes upregulated in the AtERF1 overexpression plants can be assigned to heat, drought, salt and JA responses, respectively, but unlike for biotic stress, these genes are activated through binding of ERF1 to the dehydration response element (DRE) rather than to the GCC element (Cheng et al., 2013). For the salt stress response in tobacco, JA connects not only with ET, through a jasmonate responsive tomato ERF (*JERF1*), but also with ABA signaling (Zhang et al., 2004). Also a second tomato ERF (*SlERF.B.3*) is linked with the response to salt and cold stress (Klay et al., 2014).

The JA crosstalk to brassinosteroids has also been documented. Brassinosteroids were shown to negatively regulate the JA inhibition of root growth, the point of convergence being the F-Box protein coronatine insensitive 1 (COI1) required for JA response (Ren et al., 2009). Inhibition of JA induced accumulation of anthocyanins by brassinazole in *Arabidopsis* represents a second example for a negative impact of brassinosteroid signaling upon the JA pathway (Peng et al., 2011). In this interaction between JA and brassinosteroids the WD-repeat/Myb/bHLH transcriptional complexes were implicated (Qi et al., 2011). Concordance was shown between anthocyanins and drought tolerance in rice and *Arabidopsis* (Basu et al., 2010; Sperdouli and Moustakas, 2012), thus implicating a role for JA. Divi et al. (2010) reported on the crosstalk between brassinosteroids, ET, SA, JA, and ABA using mutants in the respective phytohormone biosynthesis pathways. Recently the same authors studied the brassinosteroids-mediated stress tolerance and found distinct molecular signatures of ABA and JA (Divi et al., 2015).

Nitric oxide has emerged as a major signal affecting the JA, SA, and ET signaling in biotic stress response, and has meanwhile also been shown to act in abiotic stress responses as well (Wang et al., 2006; Song et al., 2008; Huang et al., 2009; Zhang et al., 2011). In this context, the role of NO in stomatal aperture has attracted most attention, because guard cells have emerged as convenient model system to dissect signaling cascades. The first tier of signaling is formed by pH, ROS, free Ca^2+^, and phospholipid activating the next layer of signaling by complex interactions of these primary signals with ABA, ET, JA, and NO (Gayatri et al., 2013). By treatment with NG-nitro-L-arginine methyl ester (L-NAME), an inhibitor of NO synthase (Xin et al., 2005), jasmonate-induced stomatal closure could be modulated indicating that NO acts downstream of JA. Like in most cases of signal crosstalk, specific roles, timing and convergence points of NO and JA signaling are still not characterized, which would be a precondition for strategies to promote plant tolerance to abiotic stress.

## Protein Post-Translational Modification and Jasmonates Crosstalk

Post-translational modifications (PTM) regulate many proteins critical for JA biosynthesis and their signaling crosstalk with other phytohormones. Among the various possible protein modifications, protein phosphorylation, ubiquitination and SUMOylation have been in focus for these pathways. For example, PPS3 the potato homolog of *Arabidopsis* JAI3/JAZ3, is phosphorylated by StMPK1, the MPK6 homolog of *Arabidopsis* MAP kinase (Katou et al., 2005). Dephosphorylation of proteins involved in regulating JA content has also been shown (Schweighofer et al., 2007).

In JA signaling, an important role is played by the JAZ and the DELLA proteins, both of which are ubiquitinated in the presence of jasmonates and gibberellins, respectively, and undergo proteasome-mediated degradation. JAZ degradation leads to transcriptional activation and DELLA degradation leads to repression of the JA responsive genes by MYC2 (Figure 2). Recently, SUMOylation of DELLA was shown to sustain DELLA-mediated inhibition of growth under stress (Conti et al., 2014). SUMOylated DELLAs bind to the SUMO interacting motif of the GA receptor GID1 whose sequestration leads to an accumulation of non-SUMOylated DELLAs. It remains to be seen if in such conditions the SUMOylated or non-SUMOylated content of the DELLAs leads to their altered interaction with the JAZ proteins and influences the activity of the JA responsive genes.

The identification of *auxin-resistant1* (*axr1*) mutants with altered jasmonate responsive gene expression during screening for mutants resistant to MeJA- and auxin-mediated growth inhibition indicated JA-auxin crosstalk (Tiryaki and Staswick, 2002). *AXR1* encodes an enzyme that activates a second small protein related to ubiquitin (RUB1; Nedd8 in mammals). Therefore, both jasmonate and auxin signal transduction depends on small modifier proteins (Lorenzo and Solano, 2005).

Salicylic acid signaling is controlled by a SUMO E3 ligase (SIZ1) whereby *siz1* mutants of *Arabidopsis* accumulate SA (Lee et al., 2007). SIZ1 is also known to regulate drought responses without the involvement of DREB2A and ABA, with the expression of nearly 10% of the drought inducible genes being mediated by SIZ1 (Catala et al., 2007). These genes also include some for JA responses. Down-regulation of 11 genes of the brassinosteroid biosynthesis and signaling pathway was noted through the genome-wide expression analysis of the *siz1* mutants. This suggested an important role for protein SUMOylation in these pathways (Catala et al., 2007).

Interestingly, NO content is highly influenced by the SUMOylation of the nitrate reductases (NRs) NIA1 and NIA2, and SUMOylation substantially increases the activity of the two NRs (Park et al., 2011). Finally, SIZ1, the SUMO E3 ligase, is also involved in copper homeostasis putatively through regulating the metal transporters YELLOW STRIPE LIKE (*YSL1* and *YSL3*; Chen et al., 2011), which affects ethylene perception (Burkhead et al., 2009).

Apparently, SUMO conjugation-deconjugation to proteins plays an important role in abiotic stress (Castro et al., 2012; Raorane et al., 2013). This section implies that a number of effects of SUMO on abiotic stress may be mediated through affecting phytohormone biosynthesis and signaling crosstalk. When phytohormone crosstalk is so intricately linked that spatio-temporal signatures of active hormone content are the defining features, rather than overall content, PTM of the proteins involved in the various crosstalk routes becomes a regulatory feature that is likely as important as their transcription and translation *per se*. For a holistic understanding of JAs as emerging molecules of importance in abiotic stress tolerance, not only their crosstalk to other phytohormones but also the protein PTM aspects must be deeply explored.

## Can Fine-Tuning of the Jasmonate Pathway Lead to Abiotic Stress Tolerance?

Plants can resist abiotic stresses through several distinct mechanisms, but the traits associated with resistance mechanisms are multigenic, often converging on genes shared by different stresses. Under stress conditions, the interactions happening between signaling pathways and their biological significance still remain unclear. As of now, these pathways are getting better resolved due to the evolution of new tools that allow the exploration of the physiological, genetic, and biochemical basis of such processes. The use of genomic, proteomic, and metabolomic approaches is gaining grounds not only in model plants such as *Arabidopsis* but also in crops such as rice. These investigative strategies will unravel new crosstalks between the different classes of stress hormones (for review, see, e.g., Kazan, 2015).

Keeping in view the immense losses caused by adverse environmental conditions like drought or salinity, there is an immediate need to develop new crop varieties with better adaptability or enhanced tolerance. Till date only a handful of labs around the world have been able to show a direct relation between JA functioning as a stress hormone in salinity and drought tolerance. The examples discussed here present a substantial reason to suggest a central role of JA as a hormone for these stress responses. More research focus in this direction will help to explain various drought stress responses and provide powerful tools for improving drought tolerance in plants and to develop new drought tolerant varieties. However, it will be a big challenge to manipulate JA biosynthesis or signaling without giving rise to negative side effects commonly associated with reduced jasmonate function such as reduced fertility and enhanced sensitivity to pathogens. Finding the critical nodes in the phytohormone biosynthetic pathways, whose manipulation can be useful for stress tolerance without the associated penalties; will largely define the level of success in utilizing these pathways for breeding stress tolerant crop varieties.

## Author Contributions

MR, RD, MH, BM, AK, and PN drafted the manuscript. BM and MR designed the figures. MR, RD, AK, and PN revised the manuscript.

### Conflict of Interest Statement

The authors declare that the research was conducted in the absence of any commercial or financial relationships that could be construed as a potential conflict of interest.
